# Reference Quality Genome Assemblies of Three *Parastagonospora nodorum* Isolates Differing in Virulence on Wheat

**DOI:** 10.1534/g3.117.300462

**Published:** 2017-12-12

**Authors:** Jonathan K. Richards, Nathan A. Wyatt, Zhaohui Liu, Justin D. Faris, Timothy L. Friesen

**Affiliations:** * Department of Plant Pathology, North Dakota State University, Fargo, North Dakota 58102; † Genomics and Bioinformatics Program, North Dakota State University, Fargo, North Dakota 58102; ‡ Cereal Crops Research Unit, Red River Valley Agricultural Research Center, United States Department of Agriculture-Agricultural Research Service (USDA-ARS), Fargo, North Dakota 58102

**Keywords:** *Parastagonospora nodorum*, genome sequencing, RNAseq, wheat, effector, Genome Report

## Abstract

*Parastagonospora nodorum*, the causal agent of Septoria nodorum blotch in wheat, has emerged as a model necrotrophic fungal organism for the study of host–microbe interactions. To date, three necrotrophic effectors have been identified and characterized from this pathogen, including SnToxA, SnTox1, and SnTox3. Necrotrophic effector identification was greatly aided by the development of a draft genome of Australian isolate SN15 via Sanger sequencing, yet it remained largely fragmented. This research presents the development of nearly finished genomes of *P. nodorum* isolates Sn4, Sn2000, and Sn79-1087 using long-read sequencing technology. RNAseq analysis of isolate Sn4, consisting of eight time points covering various developmental and infection stages, mediated the annotation of 13,379 genes. Analysis of these genomes revealed large-scale polymorphism between the three isolates, including the complete absence of contig 23 from isolate Sn79-1087, and a region of genome expansion on contig 10 in isolates Sn4 and Sn2000. Additionally, these genomes exhibit the hallmark characteristics of a “two-speed” genome, being partitioned into two distinct GC-equilibrated and AT-rich compartments. Interestingly, isolate Sn79-1087 contains a lower proportion of AT-rich segments, indicating a potential lack of evolutionary hotspots. These newly sequenced genomes, consisting of telomere-to-telomere assemblies of nearly all 23 *P. nodorum* chromosomes, provide a robust foundation for the further examination of effector biology and genome evolution.

*Parastagonospora nodorum* is the causal agent of Septoria nodorum blotch in wheat. *P. nodorum* produces small, secreted proteins known as necrotrophic effectors (NEs) to infect its host by triggering programmed cell death (PCD), resulting in NE-triggered susceptibility. This interaction has been classified as inverse gene-for-gene (Friesen *et al.* 2006), in contrast to the classical gene-for-gene model (Flor 1956), since PCD is to the advantage of the pathogen rather than the host (Friesen *et al.* 2006). Within *P. nodorum*, NEs have been observed to be high in relative cysteine content, promoting protein stability (Liu *et al.* 2009, 2012, 2016). Additionally, these NEs are often flanked by repetitive elements and exhibit complete gene absence in avirulent isolates (Oliver *et al.* 2012). Three *P. nodorum* effector genes have been cloned and characterized, including *SnToxA*, *SnTox1*, and *SnTox3* (Friesen *et al.* 2006; Liu *et al.* 2009, 2012).

Knowledge of genomic architecture and gene annotations of plant pathogenic fungi can facilitate effector identification, as well as enable the study of pathogen evolution via comparative analyses (Gibriel *et al.* 2016). The use of short-read technologies generally results in largely fragmented genome assemblies due to their inability to span repetitive regions. Long-read sequencing technologies, such as single-molecule real-time (SMRT) sequencing, have the ability to bridge fragmented genomes by producing average read lengths of >10 kb, essentially sequencing through repetitive elements (Thomma *et al.* 2016; Rhoads and Au 2015). The development of complete genomes directly benefits efforts toward effector identification, as previously identified effectors have often been discovered in the rapidly changing, repetitive compartments of the genome (Friesen *et al.* 2006; Gout *et al.* 2006; Thomma *et al.* 2016).

Hane *et al.* (2007) reported the first genome sequence of *P. nodorum* isolate SN15 (Australia). Synthesized via Sanger sequencing, the initial draft genome was assembled into 107 nuclear scaffolds, totaling ∼37 Mb and predicted at least 10,762 genes (Hane *et al.* 2007). Resequencing of SN15, as well as isolates Sn4 and Sn79-1087, with Illumina short-read technology and the addition of RNAseq and proteomics datasets improved the initial draft genome to 91 scaffolds and updated SN15 gene annotations to 13,569 total genes (Syme *et al.* 2013, 2016). Here we report the synthesis of three reference quality genome sequences of the *P. nodorum* isolates LDN03-Sn4 (hereafter referred to as Sn4), Sn2000, and Sn79-1087 (Faris *et al.* 2011; Liu *et al.* 2004b; Friesen *et al.* 2006) using SMRT sequencing technology, resulting in telomere-to-telomere assemblies of nearly every chromosome of each isolate. Additionally, RNAseq data derived from eight time points, including one culture and seven *in planta* infection time points of isolate Sn4, provided a robust framework for gene annotation. Isolates Sn4 and Sn2000 harbor different complements of NEs (Bertucci *et al.* 2014), whereas isolate Sn79-1087 is avirulent (Friesen *et al.* 2006). The development of the polished genomes and subsequent annotations presented here will expedite effector identification, as well as enable the comparison of genome architecture of these and other *P. nodorum* isolates.

## Materials and Methods

### Biological materials and DNA extraction

Tissue of *P. nodorum* isolates Sn4 (Liu *et al.* 2009), Sn2000 (Liu *et al.* 2004b), and Sn79-1087 (Friesen *et al.* 2006) were grown in 75 ml of Fries media (Liu *et al.* 2004a) for 3 d from dried mycelial plugs. Fungal tissue was rinsed with sterile distilled H_2_O and subsequently lyophilized. Lysis buffer was prepared by combining 6.5 ml of buffer A (350 mM sorbitol, 5 mM EDTA, and 100 mM Tris-Cl), 6.5 ml of buffer B (50 mM EDTA, 2000 mM NaCl, 200 mM Tris-Cl, and 2% CTAB), 2.6 ml of buffer C (5% *N*-lauroylsarcosine), and 1.75 ml of 1% polyvinylpyrrolidone. A total of 500 mg of lyophilized tissue was added to a 50 ml conical tube (two tubes per isolate), followed by homogenization in 25 ml lysis buffer and 150 µl RNase A (20 mg/ml). The samples were incubated for 30–45 min at 65°, mixing every 15 min. A volume of 8.25 ml of 5 M potassium acetate (pH 7.5) was added to each tube, mixed by inversion, and incubated on ice for 30 min. Samples were centrifuged for 20 min at ∼3100 × *g* at 4°, and the aqueous phase was transferred to a new 50 ml conical tube. A 0.1 vol of 3 M sodium acetate (pH 5.2) and equal volume of room temperature isopropyl alcohol were added to each tube, mixed by inversion, and incubated at room temperature for 5 min. Precipitated DNA was collected with a glass hook and subsequently rinsed with 5 ml of freshly prepared 70% ethanol. The ethanol was removed, the DNA was rinsed again, and transferred to a new 1.5 ml tube. Excess ethanol was removed by pipetting and the DNA pellet was freeze-dried for 5–10 min. DNA was resuspended in 500 µl H_2_O and incubated at 65° for 30 min, followed by incubation at 4° overnight. Samples were then centrifuged at 2000 × *g* for 2 min, and the supernatants of samples from the same isolate were combined into new 15 ml conical tubes using a large bore pipette tip. High-molecular-weight DNA was then purified using the Qiagen Genomic-Tip 100/G kit according to the manufacturer’s recommended protocol.

### SMRT sequencing

SMRT sequencing libraries of *P. nodorum* isolates Sn4, Sn2000, and Sn79-1087 were prepared and sequenced from isolated high-molecular-weight DNA at the Mayo Clinic Molecular Biology Core (Rochester, MN). Libraries were subsequently sequenced on the PacBio RSII instrument with a 20-kb size-selected library and P6-C4 chemistry. A total of nine SMRT cells were sequenced for *P. nodorum* isolate Sn4, and four SMRT cells were sequenced for both isolates Sn2000 and Sn79-1087.

### RNA extraction, RNAseq library preparation, and sequencing

Cultures and inoculum of *P. nodorum* isolate Sn4 were prepared as previously described (Liu *et al.* 2004b). Seeds of wheat line ND495 were sown into containers surrounded by a border of wheat line Alsen and grown under greenhouse conditions for ∼2 wk. Inoculations were conducted as described by Liu *et al.* (2004b). Following inoculation, plants were moved to a mist chamber in the light at 100% humidity for 24 hr and were subsequently moved to a growth chamber at 21° with a 12 hr photoperiod. Tissue was collected at 1, 2, 3, 5, 7, 9, and 14 d postinoculation (dpi). Tissue was immediately flash frozen in liquid nitrogen and stored at −80° until RNA extraction. Liquid cultures of isolates Sn4, Sn2000, and Sn79-1087 were prepared by incubating five dried mycelial plugs in 75 ml of Fries media (Liu *et al.* 2004a) for 14 d. Tissue was harvested, rinsed, and immediately flash frozen in liquid nitrogen and stored at −80°. A total of three biological replicates were collected at each time point. mRNA from each sample was isolated utilizing the mRNA Direct Kit (Thermo Fisher Scientific) following the manufacturer’s protocol. Strand nonspecific RNAseq libraries were prepared with the Illumina TruSeq RNA Sample Preparation v2, using purified mRNA as input, according to the manufacturer’s recommended protocol. Quality and fragment size distribution of the prepared RNAseq libraries was determined using an Agilent DNA chip on a bioanalyzer (Agilent, Santa Clara, CA). Libraries were subsequently sequenced on an Illumina NextSeq at the USDA-ARS Small Grains Genotyping Center (Fargo, ND) to produce 150 bp single-end reads.

### De novo genome assembly

Raw SMRT sequencing reads were input into the Pacific Biosciences SMRTportal software installed on a local Linux machine. Using the HGAPv3 protocol within the SMRTportal software, raw reads were trimmed, corrected, and *de novo* assembled under default parameters with a genome size estimate of 37.2 Mb (Chin *et al.* 2013). Within the PacBio SMRTportal HGAPv3 protocol, assemblies were polished utilizing the previously error-corrected reads using Quiver. All assembled nuclear contigs <150 kb in size were discarded. Identification of the contig corresponding to the mitochondrial genome was facilitated via BLAST searches using the assembled contigs as queries against the previously assembled mitochondrial genome of *P. nodorum* SN15 (Hane *et al.* 2007).

The genomes of various filamentous fungal pathogens exhibit a two-speed genome, where two distinct genomic compartments exist, consisting of marked differences in GC content (Dong *et al.* 2015). The genome architecture of *P. nodorum* isolates Sn4, Sn2000, and Sn79-1087 were examined with regard to GC content using OcculterCut with default settings (Testa *et al.* 2016).

Synteny analysis was conducted in SyMAP v4.2 (Soderlund *et al.* 2011). Sn4 nuclear contigs were sorted from largest to smallest and used as a reference for alignment. Nuclear contigs of isolates Sn2000 and Sn79-1087 were named based on their syntenic relationship with contigs from isolate Sn4.

### Gene annotation

RNAseq reads from all sequenced time points were assessed for quality using FastQC (Andrews 2010). Sequencing reads were trimmed for quality and the presence of adapter sequences using trimmomatic (Bolger *et al.* 2014). Trimmed reads from all time points and replicates were bulked and aligned to the Sn4 reference genome using Hisat2, specifying a maximum intron length of 3000 bp and the remaining options as default values (Pertea *et al.* 2016). The RNAseq-derived transcript structure and genomic coordinates were obtained using StringTie with default settings (Pertea *et al.* 2016), and transcript sequences were extracted using “gffread.” RNAseq-derived transcripts were used as EST evidence coupled with the previously annotated protein sequence from *P. nodorum* isolate SN15 (Syme *et al.* 2016), as well as *ab initio* gene prediction via GeneMark-ES (Lomsadze *et al.* 2005) and SNAP (Korf 2004) in the MAKER genome annotation pipeline (Holt and Yandell 2011) to produce the final gene annotation. Coordinates of RNAseq-derived transcript alignments were obtained from the MAKER output and used to calculate the number of genes supported by RNAseq evidence using bedtools “coverage” (Quinlan and Hall 2010). Annotations of the Sn2000 and Sn79-1087 genomes were conducted *in silico* using GeneMark-ES and the previously trained SNAP prediction software within the MAKER genome annotation pipeline. All annotated proteins from each isolate were analyzed with SignalP 4.0 (Petersen *et al.* 2011) to predict the presence of secretion signals. Predicted secreted proteins were then analyzed by EffectorP (Sperschneider *et al.* 2015) to determine the abundance of predicted effector proteins present in each isolate. Gene annotation completeness was assessed in each isolate using BUSCO v3 to determine the presence of conserved, single-copy orthologs from the Ascomycota lineage (Simão *et al.* 2015). Using the annotated protein sequences from isolates Sn4, Sn2000, Sn79-1087, and SN15, orthologous proteins were clustered using the GET_HOMOLOGS software (Contreras-Moreira and Vinuesa 2013) to determine a core set of *P. nodorum* proteins.

Secreted protein sequences from genes found in an AT-rich expansion on contig 10, as well as on dispensable contig 23, were subjected to BLASTP searches of the NCBI nonredundant Ascomycota protein database. *P. nodorum* proteins were considered conserved if a homologous protein was found with an *e*-value cutoff of 1 × 10^−5^ and query coverage >50%.

### Data availability

Assembled genome sequences and accompanying gene annotations of *P. nodorum* isolates Sn4, Sn2000, and Sn79-1087 are deposited in the NCBI database under BioProject accession number PRJNA398070.

## Results and Discussion

### Sequencing and de novo genome assembly

SMRT sequencing of *P. nodorum* isolates Sn4, Sn2000, and Sn79-1087 resulted in 485,091, 366,428, and 354,610 filtered reads per library, respectively. This corresponded to ∼5359.9–5484.8 Mb of the genomic sequence (Table 1). The average read lengths of the sequencing libraries ranged from 11,134 to 15,115 bp postfiltering. The utilization of long-read sequencing technology enabled the assembly of nearly every chromosome into telomere-to-telomere genomic contigs. The genome assemblies of Sn4, Sn2000, and Sn79-1087 consisted of 21, 19, and 20 contigs harboring telomeric repeats on both ends, respectively (Table 1). With the total number of nuclear contigs of each genome assembly ranging from 22 (Sn79-1087) to 24 (Sn4 and Sn2000), these genome assemblies greatly improve upon the previous Sanger-sequenced and Illumina resequenced *P. nodorum* isolate SN15 assembly, which currently consists of 91 scaffolds (Syme *et al.* 2016). Additionally, these assemblies bridged many gaps in the genomes of isolates Sn4 and Sn79-1087, which were previously sequenced with short-read Illumina technology and remained highly fragmented, consisting of 2559 and 3132 contigs >1 kb in length (Syme *et al.* 2013).

**Table 1:** SMRT sequencing and assembly statistics

	Sn4	Sn2000	Sn79-1087
Sequencing reads	485,091	366,428	354,610
Total sequenced bases	5,400,955,164	5,484,796,333	5,359,857,877
Average read length	11,134	14,968	15,115
Nuclear contigs	24	24	22
Nuclear contigs with both telomeres	21	19	20
Nuclear genome (bp)	37,694,868	37,459,375	34,991,254
Mitochondrial genome (bp)	75,092	68,589	68,282
L50 (contigs)*^a^*	9	9	8
L90 (contigs)*^b^*	20	20	19
N50 (bp)*^c^*	1,657,153	1,711,973	1,583,228
N90 (bp)*^d^*	1,090,035	1,118,796	1,122,469

a Smallest number of contigs whose length equals 50% of the genome assembly.

b Smallest number of contigs whose length equals 90% of the genome assembly.

c Length of the smallest contig in an ordered set of contigs corresponding to 50% of the assembly length.

d Length of the smallest contig in an ordered set of contigs corresponding to 90% of the assembly length.

A total of 10 and seven assembled contigs of isolates Sn2000 and Sn79-1087, respectively, were discarded due to contig length (<150,000 bp). These contigs were annotated as containing large proportions of repetitive sequences and likely failed to assemble into larger contigs due to the repetitive content. Additionally, the contigs were devoid of gene content, with the exception of two *ab initio* predicted genes in the Sn2000 genome.

### Synteny analysis

**Figure 1:**
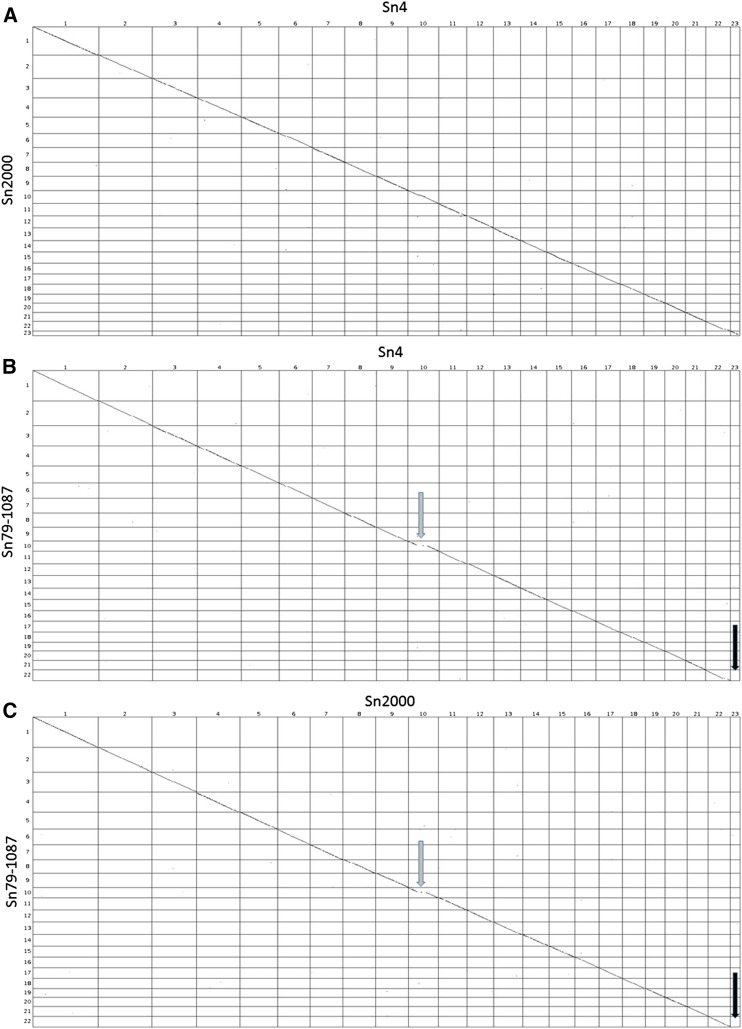
Dot plots illustrating whole-genome alignments of *P. nodorum* isolates Sn4 and Sn2000 (A), Sn4 and Sn79-1087 (B), and Sn2000 and Sn79-1087 (C). Black arrows indicate the complete absence of contig 23 in the genome of isolate Sn79-1087. Gray arrows highlight a large expansion present in contig 10 of isolates Sn4 and Sn2000 compared with Sn79-1087.

The long-read sequencing technology and subsequent high-quality genome assemblies enabled a macrosyntenic comparison of all 23 *P. nodorum* chromosomes. A total of 21 contigs in the isolate Sn4 assembly represent fully sequenced chromosomes, as telomeric repeats were detected at both ends of the contigs. Sn4 contigs 22.1 and 22.2 were joined via syntenic evidence with Sn2000 and Sn79-1087, and were subsequently merged to form Sn4 contig 22 (Figure 1, A and B). Also, Sn2000 contigs 15.1 and 15.2 were joined via alignment with Sn4 and Sn79-1087 and were merged to form Sn2000 contig 15 (Figure 1, A and C). An ∼500 kb expansion was observed in contig 10 of Sn4 and Sn2000, corresponding to an AT-rich region containing 62 genes in Sn4, as well as a high level of repetitive DNA sequences (Figure 1, B and C). Five of the genes within this AT-rich region encode predicted secreted proteins, but none are predicted to be effectors. These proteins range in size from 14.56 to 50.47 kDa, have cysteine content ranging from 0 to 1.75%, and have homologs in other Ascomycota genera (Supplemental Material, Table S1).

Additionally, Sn4 contig 23—a fully sequenced chromosome of 476,058 bp in length, harboring 126 annotated genes—was observed to be completely absent from isolate Sn79-1087, but present in the Sn2000 genome (Figure 1, B and C). A total of seven genes on this contig encoded predicted secreted proteins, including one predicted effector. These protein sizes ranged from 11.84 to 54.17 kDa and contained varying levels of cysteine residues (0–3.33%). Additionally, four of these secreted proteins were found only in the annotated genes of *P. nodorum* and had no known homologs in other Ascomycetes (Table S1). As isolate Sn79-1087 is avirulent on cultivated wheat, the genes on this chromosome may be interesting targets for their potential roles in pathogenicity or virulence. However, these genes are not critical to pathogenicity based on the high level of virulence observed when Sn79-1087 was transformed with any of the three *P. nodorum* cloned NE genes (Friesen *et al.* 2006; Liu *et al.* 2009, 2012).

### Transcript assembly and gene annotation

Using transcript evidence derived from eight RNAseq time points (culture and 1, 2, 3, 5, 7, 9, and 14 dpi) and previously annotated *P. nodorum* protein sequences (Syme *et al.* 2016), along with trained *ab initio* gene predictors, a total of 13,379 genes were annotated in the Sn4 genome, including 9415 genes supported by RNAseq reads throughout the entire length of the gene (Table 2). Gene annotation using gene prediction software trained using isolate Sn4 resulted in 13,532 and 13,294 genes in the Sn2000 and Sn79-1087 genomes, respectively (Table 2). The total number of genes identified in the three newly sequenced *P. nodorum* isolates compares similarly with the previously annotated isolate SN15 containing 13,569 genes (Syme *et al.* 2016). Slight differences in the number of annotated genes are likely due to the presence/absence variations between the isolates, as well as differences in the software used in analysis. Additionally, the prediction of the secretome of each isolate resulted in the identification of 1361, 1328, and 1247 proteins harboring a predicted secretion signal in isolates Sn4, Sn2000, and Sn79-1087, respectively (Table 2). Of these predicted secreted proteins, a total of 287, 281, and 237 proteins in isolates Sn4, Sn2000, and Sn79-1087, respectively, are predicted effectors (Table 2). Previously cloned *P. nodorum* effector genes *SnToxA* and *SnTox1* were identified as predicted effectors present in the genomes of isolates Sn4 and Sn2000, whereas previously characterized *SnTox3* was only identified in isolate Sn4, in agreement with prior research (Friesen *et al.* 2006; Liu *et al.* 2009, 2012) As these effectors are present in virulent isolates but absent in avirulent isolates such as Sn79-1087, genes exhibiting this type of variation may be targeted for further characterization as effector candidates.

**Table 2:** Annotated gene properties

	Sn4	Sn2000	Sn79-1087
Annotated genes	13,379	13,532	13,294
Mean gene length (bp)	1402.0	1376.8	1384.1
Mean exon count	2.7	2.6	2.6
Predicted secreted proteins*^a^*	1361	1328	1247
Predicted effector proteins*^b^*	287	281	237
Conserved Ascomycota orthologs (%)*^c^*	97.3	97.5	97.9

a Proteins harboring predicted signal sequence via SignalP (Petersen *et al.* 2011).

b Secreted proteins predicted to be effectors via EffectorP (Sperschneider *et al.* 2015)

c Proportion of 1315 conserved Ascomycota orthologous genes present in annotated gene set as determined via BUSCO (Simão *et al.* 2015)

BUSCO was used to assess the completeness of the gene annotations from each *P. nodorum* isolate. Using the presence of 1315 conserved, single-copy orthologs from the Ascomycota phylum as criteria, the gene annotations of Sn4, Sn2000, and Sn79-1087 were estimated to be 97.3, 97.5, and 97.9% complete, respectively (Table 2). Protein clustering using the software GET_HOMOLOGS (Contreras-Moreira and Vinuesa 2013) identified 10,637 clusters of orthologous proteins between the annotated proteomes of *P. nodorum* isolates Sn4, Sn2000, Sn79-1087, and SN15. These results are similar to those described by Syme *et al.* (2016) and likely represent a conserved core set of *P. nodorum* genes.

### Compartmentalization of GC content

**Figure 2:**
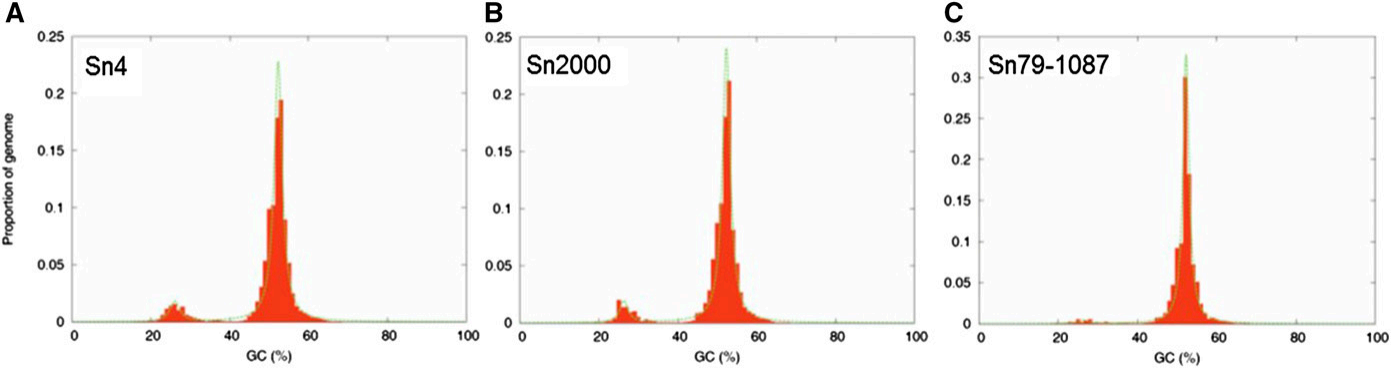
Distribution of GC content in the genomes of Sn4 (A), Sn2000 (B), and Sn79-1087 (C). GC content (%) is illustrated on the *x*-axis and the proportion of the genome (0–1.00) is shown on the *y*-axis.

Comparative genome analysis of fungal plant pathogens has revealed the pattern of a two-speed genome in various species, consisting of GC-equilibrated, gene-dense compartments and repeat-rich, gene-sparse compartments (Dong *et al.* 2015). Genes harbored in repeat-rich regions have been observed to undergo higher rates of positive selection, indicating these compartments may be rapidly changing (Raffaele *et al.* 2010). Repeat-induced point mutation, a genome defense mechanism against duplication events, may be a driving factor in the development of these AT-rich areas and aid the rapid evolution of genes within these regions (Lo Presti *et al.* 2015; Testa *et al.* 2016). Analysis of the GC content of the *P. nodorum* isolates Sn4, Sn2000, and Sn79-1087 revealed the presence of a bipartite genome architecture (Figure 2 and Table 3). The AT-rich regions comprised ∼9.0 and 8.5% of the genomes of isolates Sn4 and Sn2000, respectively (Figure 2, A and B and Table 3). Interestingly, the portion of the genome of isolate Sn79-1087 corresponding to elevated AT content was considerably lower, only accounting for 2.8% of the nuclear genome (Figure 2C and Table 3). Additionally, a lower gene density of 3.1 genes/Mbp within these regions was also observed, compared with 12.6 and 15.9 genes/Mbp within the AT-rich regions of isolates Sn4 and Sn2000, respectively (Table 3). As Sn79-1087 is avirulent on cultivated wheat, these results indicate that this isolate may lack the evolutionary active regions of the genome harboring putative effectors.

**Table 3:** Genomic GC contents

	Sn4	Sn2000	Sn79-1087
High GC content range (%)*^a^*	34.3–100.0	34.2–100.0	32.7–100.0
High GC content genome proportion (%)*^b^*	91.0	91.5	97.2
High GC content peak (%)*^c^*	52.3	52.3	52.2
Low GC content range (%)*^d^*	0.0–34.3	0.0–34.2	0.0–32.7
Low GC content genome proportion (%)*^e^*	9.0	8.5	2.8
Low GC content peak (%)*^f^*	26.0	26.3	26.4
Gene density in high GC regions (genes/Mbp)*^g^*	389.0	394	391
Gene density in low GC regions (genes/Mbp)*^h^*	12.6	15.9	3.1

Genomic GC content attributes as derived from analysis via OcculterCut (Testa *et al.* 2016) indicating a two-speed genome.

a Range of GC content in the elevated GC regions of the genome.

b Proportion of the genome containing a relatively higher GC content.

c Peak GC content within the high GC genomic compartment.

d Range of GC content in the relatively lower GC regions of the genome.

e Proportion of the genome containing a relatively lower GC content.

f Peak GC content within the low GC genomic compartment.

g Density of annotated genes within the relatively high GC regions of the genome.

h Density of annotated genes within the relatively low GC regions of the genome.

These results are similar to the previously sequenced *P. nodorum* isolate SN15, which was shown to also have a compartmentalized genome, with ∼6.64% of the genome comprised of AT-rich segments and ∼0.8 genes/Mbp within these regions (Testa *et al.* 2016). The increased AT-rich genome proportion in isolates Sn4 and Sn2000 in comparison with SN15, as well as the increased gene density within these areas, is likely due to the long-read sequencing technology used and subsequent ability to assemble repeat-rich regions of the genome.

### Conclusions

High-quality reference genomes and gene annotations of *P. nodorum* isolates Sn4, Sn2000, and Sn79-1087 were developed via long-read sequencing technology, assembly, and integration of robust transcriptomics datasets spanning multiple developmental and lifecycle stages of *P. nodorum*. These polished genomes represent a telomere-to-telomere assembly of nearly every chromosome of the aforementioned isolates, presenting a significant improvement over the previous fragmented draft genomes. Comparative analyses reveal chromosome-level polymorphism, as evidenced by the absence of contig 23 from isolate Sn79-1087, as well as regions of genome expansion or deletion. Additionally, the genome architecture of isolate Sn79-1087 exhibits a lower genome proportion of AT-rich regions, potentially indicating the lack of effector hotspots. This research illustrates the utility of long-read sequencing technology and genome plasticity of *P. nodorum*, and also enables further investigation of the genome evolution and effector biology of this necrotrophic pathogen.

## Supplementary Material

Supplemental material is available online at www.g3journal.org/lookup/suppl/doi:10.1534/g3.117.300462/-/DC1.

## References

[bib1] Andrews, S., 2010 FastQC: a quality control tool for high throughput sequence data. Available at: http://www.bioinformatics.babraham.ac.uk/projects/fastqc.

[bib2] Bertucci M. Brown-Guedira G. Murphy J. P. Cowger C., 2014 Genes conferring sensitivity to *Stagonospora nodorum* necrotrophic effectors in Stagonospora nodorum blotch-susceptibile U.S. wheat cultivars. Plant Dis. 98: 746–753. 10.1094/PDIS-08-13-0820-RE 30708627

[bib3] Bolger A. M. Lohse M. Usadel B., 2014 Trimmomatic: a flexible trimmer for Illumina sequence data. Bioinformatics 30: 2114–2120. 24695404 10.1093/bioinformatics/btu170 PMC4103590

[bib4] Chin C. S. Alexander D. H. Marks P. Klammer A. A. Drake J., 2013 Nonhybrid, finished microbial genome assemblies from long-read SMRT sequencing data. Nat. Methods 10: 563–569. 23644548 10.1038/nmeth.2474

[bib5] Contreras-Moreira B. Vinuesa P., 2013 GET_HOMOLOGUES, a versatile software package for scalable and robust microbial pangenome analysis. Appl. Environ. Microbiol. 79: 7696–7701. 24096415 10.1128/AEM.02411-13 PMC3837814

[bib6] Dong S. Raffaele S. Kamoun S., 2015 The two-speed genomes of filamentous pathogens: waltz with plants. Curr. Opin. Genet. Dev. 35: 57–65. 26451981 10.1016/j.gde.2015.09.001

[bib7] Faris J. D. Zhang Z. Rasmussen J. B. Friesen T. L., 2011 Variable expression of the *Stagonospora nodorum* effector SnToxA among isolates is correlated with levels of disease in wheat. Mol. Plant Microbe Interact. 24: 1419–1426. 21770771 10.1094/MPMI-04-11-0094

[bib8] Flor H. H., 1956 The complementary genic systems in flax and flax rust. Adv. Genet. 8: 29–54.

[bib9] Friesen T. L. Stukenbrock E. H. Liu Z. Meinhardt S. Ling H., 2006 Emergence of a new disease as a result of interspecific virulence gene transfer. Nat. Genet. 38: 953–956. 16832356 10.1038/ng1839

[bib10] Gibriel H. A. Y. Thomma B. P. H. J. Seidl M. F., 2016 The age of effectors: genome-based discovery and applications. Phytopathology 106: 1206–1212. 27050568 10.1094/PHYTO-02-16-0110-FI

[bib11] Gout L. Fudal I. Kuhn M. L. Blaise F. Eckert M., 2006 Lost in the middle of nowhere: the AvrLm1 avirulence gene of the Dothideomycete *Leptosphaeria maculans*. Mol. Microbiol. 60: 67–80. 16556221 10.1111/j.1365-2958.2006.05076.x

[bib12] Hane J. K. Lowe R. G. T. Solomon P. S. Tan K. Schoch C. L., 2007 Dothideomycete-plant interactions illuminated by genome sequencing and EST analysis of the wheat pathogen *Stagonospora nodorum*. Plant Cell 19: 3347–3368. 18024570 10.1105/tpc.107.052829 PMC2174895

[bib13] Holt C. Yandell M., 2011 MAKER2: an annotation pipeline and genome-database management tool for second-generation genome projects. BMC Bioinformatics 12: 491. 22192575 10.1186/1471-2105-12-491 PMC3280279

[bib14] Korf I., 2004 Gene finding in novel genomes. BMC Bioinformatics 14: 59. 10.1186/1471-2105-5-59 PMC421630 15144565

[bib15] Liu Z. Faris J. D. Oliver R. P. Tan K. Solomon P. S., 2009 SnTox3 acts in effector triggered susceptibility to induce disease on wheat carrying the *Snn3* gene. PLoS Pathog. 5: e10000581. 10.1371/journal.ppat.1000581 PMC2736379 19806176

[bib16] Liu Z. Zhang Z. Faris J. D. Oliver R. P. Syme R., 2012 The cysteine rich necrotrophic effector SnTox1 produced by *Stagonospora nodorum* triggers susceptibility of wheat lines harboring *Snn1*. PLoS Pathog. 8: e1002467. 22241993 10.1371/journal.ppat.1002467 PMC3252377

[bib17] Liu Z. Gao Y. Kim Y. M. Faris J. D. Shelver W. L., 2016 SnTox1, a *Parastagonopora nodorum* necrotrophic effector, is a dual-function protein that facilitates infection while protecting from wheat-produced chitinases. New Phytol. 211: 1052–1064. 27041151 10.1111/nph.13959

[bib18] Liu Z. H. Faris J. D. Meinhardt S. W. Ali S. Rasmussen J. B., 2004 a Genetic and physical mapping of a gene conditioning sensitivity in wheat to a partially purified host-selective toxin produced by *Stagonospora nodorum*. Phytopathology 94: 1056–1060. 18943793 10.1094/PHYTO.2004.94.10.1056

[bib19] Liu Z. H. Friesen T. L. Rasmussen J. B. Ali S. Meinhardt S. W., 2004 b Quantitative trait loci analysis and mapping of seedling resistance to Stagonospora nodorum leaf blotch in wheat. Phytopathology 94: 1061–1067. 18943794 10.1094/PHYTO.2004.94.10.1061

[bib20] Lomsadze A. Ter-Hovhannisyan V. Chernoff Y. O. Borodovsky M., 2005 Gene identification in novel eukaryotic genomes by self-training algorithm. Nucleic Acids Res. 33: 6494–6506. 16314312 10.1093/nar/gki937 PMC1298918

[bib21] Lo Presti L. Lanver D. Schweizer G. Tanaka S. Liang L., 2015 Fungal effectors and plant susceptibility. Annu. Rev. Plant Biol. 66: 513–545. 25923844 10.1146/annurev-arplant-043014-114623

[bib22] Oliver R. P. Friesen T. L. Faris J. D. Solomon P. S., 2012 *Stagonospora nodorum*: from pathology to genomics and host resistance. Annu. Rev. Phytopathol. 50: 23–43. 22559071 10.1146/annurev-phyto-081211-173019

[bib23] Pertea M. Kim D. Pertea G. M. Leek J. T. Salzberg S. L., 2016 Transcript-level expression analysis of RNA-seq experiments with HISAT, StringTie, and Ballgown. Nat. Protoc. 11: 1650–1667. 27560171 10.1038/nprot.2016.095 PMC5032908

[bib24] Petersen T. M. Brunak S. von Heijne G. Nielsen H., 2011 SignalP 4.0: discriminating signal peptides from transmembrane regions. Nat. Methods 8: 785–786. 21959131 10.1038/nmeth.1701

[bib25] Quinlan A. R. Hall I. M., 2010 BEDTools: a flexible suite of utilities for comparing genomic features. Bioinformatics 26: 841–842. 20110278 10.1093/bioinformatics/btq033 PMC2832824

[bib26] Raffaele S. Farrer R. A. Cano L. M. Studholme D. J. MacLean D., 2010 Genome evolution following host jumps in the Irish potato famine pathogen lineage. Science 330: 1540–1543. 21148391 10.1126/science.1193070

[bib27] Rhoads A. Au K. F., 2015 PacBio sequencing and its applications. Genomics Proteomics Bioinformatics 13: 278–289. 26542840 10.1016/j.gpb.2015.08.002 PMC4678779

[bib28] Simão F. A. Waterhouse R. M. Ioannidis P. Kriventseva E. V. Zdobnov E. M., 2015 BUSCO: assessing genome assembly and annotation completeness with single-copy orthologs. Bioinformatics 31: 3210–3212. 26059717 10.1093/bioinformatics/btv351

[bib29] Soderlund C. Bomhoff M. Nelson W. M., 2011 SyMAP v3.4: a turnkey synteny system with application to plant genomes. Nucleic Acids Res. 39: e68. 21398631 10.1093/nar/gkr123 PMC3105427

[bib30] Sperschneider J. Gardiner D. M. Dodds P. N. Tini F. Covarelli L., 2015 EffectorP: predicting fungal effector proteins from secretomes using machine learning. New Phytol. 210: 743–761. 26680733 10.1111/nph.13794

[bib31] Syme R. A. Hane J. K. Friesen T. L. Oliver R. P., 2013 Resequencing and comparative genomics of *Stagonospora nodorum*: sectional gene absence and effector discovery. G3 (Bethesda) 3: 959–969. 23589517 10.1534/g3.112.004994 PMC3689807

[bib32] Syme R. A. Tan K. Hane J. K. Dodhia K. Stoll T., 2016 Comprehensive annotation of the *Parastagonospora nodorum* reference genome using next-generation genomics, transcriptomics, and proteogenomics. PLoS One 11: e0147221. 26840125 10.1371/journal.pone.0147221 PMC4739733

[bib33] Testa A. C. Oliver R. P. Hane J. K., 2016 OcculterCut: a comprehensive survey of AT-rich regions in fungal genomes. Genome Biol. Evol. 8: 2044–2064. 27289099 10.1093/gbe/evw121 PMC4943192

[bib34] Thomma B. P. H. J. Seidl M. F. Shi-Kunne X. Cook D. E. Bolton M. D., 2016 Mind the gap; seven reasons to close fragmented genome assemblies. Fungal Genet. Biol. 90: 24–30. 26342853 10.1016/j.fgb.2015.08.010

